# Lung function of tuberculosis patients after completion of treatment in Sidama, South Ethiopia

**DOI:** 10.3389/fmed.2025.1451861

**Published:** 2025-03-18

**Authors:** Endrias Markos Woldesemayat, Jaime H. Vera, Clea Tanner, Alemu Tamiso, Amare Assefa, Yohannes Markos Woldesenbet

**Affiliations:** ^1^College of Medicine and Health Sciences, Hawassa University, Hawassa, Ethiopia; ^2^Department of Global Health and Infection, Brighton and Sussex Medical School, University of Sussex, Brighton, United Kingdom; ^3^College of Health Sciences, Wolayta Sodo University, Soddo, Ethiopia

**Keywords:** lung function, post-TB treatment, Sidama, South Ethiopia, COPD, airway obstruction

## Abstract

**Introduction:**

Lung function impairment are commonly reported after treatment of pulmonary tuberculosis (TB). This study aimed to examine lung function parameters among adults who have undergone treatment for pulmonary TB.

**Methods:**

A comparative cross-sectional study was conducted in eight rural communities of Dale district of Sidama Region, Southern Ethiopia. The post-TB group were smear-positive TB patients who successfully completed TB treatment between 2010 and 2021, while controls were selected from the neighbors of these participants with no documented history of TB. Lung function tests were conducted using a portable spirometer, and pulmonary symptoms were assessed using the Saint Georges Respiratory Questionnaire. Obstructive pulmonary impairment was defined as the ratio of FEV1/FVC below 70% and restrictive impairment was defined as having a normal FEV1/FVC ratio with a low FEV_1_. Data were entered and analyzed using SPSS version 25 statistical package.

**Results:**

We enrolled 167 TB cases and 156 controls in the study. The median (IQR) was 38 (21) years for cases and 35 (21.75) years for controls. The median (IQR) time after completion of TB treatment was 24 (31.75) months for the TB cases. A higher proportion of post-TB participants (101/167 (60.5%; 95% confidence interval (CI), 52.9–67.6%)) than controls (63/156 (40.4%; 95% CI, 33.0–48.2%)) had obstructive impairment, *p* = 0.01. Thirty eight (22.8; 95% CI, 17.1–29.7) post-TB participants and 39 (25.0%; 95% CI, 18.9–32.3%) controls had restrictive impairment; *p* = 0.3. Post-TB participants had high risk of obstructive pulmonary impairment (adjusted hazards ratio [aOR], 2.1; 95% CI, 1.3–3.3) and chronic obstructive pulmonary diseases symptoms scores (aOR, 73.0; 95% CI, 35.3–151.2). BMI was associated with obstructive impairment, (aOR, 1.6; 95% CI, 1.0–2.6) and the post-TB participants had increased risk of any impairment (aOR, 2.2; 95% CI, 1.1–4.5).

**Conclusion:**

Post-TB participants had greater risk of pulmonary impairment and respiratory symptoms. Post-TB treatment follow-up care is suggested to quickly identify and manage pulmonary impairment.

## Introduction

Tuberculosis (TB) is a chronic infectious disease caused by mycobacterium species, it primarily affects the lungs but can also involve any body system (1). In 2021, 10.6 million new cases of TB were reported globally (2), with the treatment success rates of over 85% (2). Although, many patients who have been successfully treated for TB are living today (2), some of them continue suffering due to lung function impairment (3). Evidences confirmed that there is post-TB airflow obstruction (AFO) (4, 5). Pulmonary impairment may lead to potential health deterioration in TB patients (6); they can have cough, limited physical activity and lower health-related quality of life (7).

Air flow obstruction occurs due to lesions affecting the distal respiratory pathways during active TB. Long-term effects of TB include inflammation and destruction of the lung parenchyma, which causes scarring and fibrosis in the air way (8). There can be structural damage of airways, bronchiolar narrowing, bronchiolitis and emphysematous changes, caused by chronic or recurrent inflammation (8). These changes may remain after successful microbiological treatment (9). Air flow obstruction during TB treatment is a common manifestation of underlying chronic obstructive pulmonary disease (COPD), which mostly occurs later, during the reparative processes (6). During the active pulmonary TB treatment period, restrictive pulmonary impairment is common and may persist, resolve, or become obstructive in nature (10). AFO in patients with PTB may also be related to genetic factors, systemic inflammation response, the extent of pulmonary lesion, history of smoking and air pollution (11, 12).

Prevalence of pulmonary impairment among TB patients was 47.7% in Russia, with slightly over a third, 34.6%, of the patients having obstructive lung impairment (13). The proportion of TB patients with AFO was 21.3% in China, 30.7% in Latin America and 30.3% in Korea (12, 14). The overall prevalence of AFO in people with a history of TB was 30.7%, compared with 13.9% among those without (12). Reduced pulmonary function was reported among 28.0% of TB patients in another report (13). In African settings, the prevalence of lung function impairment reported was 45.4% with (4.1% obstructive, 36.1% restrictive) in Cameroon (15), 64.5% at week 52 of completion of TB treatment in Mozambique (16), and 45.0% in Benin (17).

Risk factors for reduced pulmonary function included past culture-positive PTB, being over 50 years old, and having recurrent TB (13). The risk of COPD was higher among people with a history of TB than among those without (18). A history of TB was also one of the risk factors of AFO (12, 14). Prior pulmonary TB, along with age, being male, asthma, and smoking were among the risk factors reported for AFO in another study (19). In participants with prior PTB, inactive TB lesions on a chest x-ray were among the risk factors of AFO (19). In Mozambique, female sex, low haemoglobin and heavy smoking were significantly associated with lung function impairment (16). In Cameroon duration of symptoms and fibrotic pattern were independent risk factors for lung function impairment (15). In a meta-analysis report, lung function impairment was varied by HIV status, geography, and across urban–rural settings (20).

Tuberculosis patients could have a chronic sequelae, which may results in lung function impairment (10). A high number of deaths following successful TB treatment were reported in the study area (21). A possible cause of these deaths and poor TB treatment outcomes could be lung function impairment. However, there are no studies on lung function impairment among TB patients who successfully completed a directly observed treatment short course (DOTS) in Sidama Region, Ethiopia, where the smear-positive TB (PTB+) case notification rate is high. The TB case notification rate reported in the study area in a previous report was 111/10^5^ populations (22). Of this, about 60% are expected to be smear-positive TB (PTB+) cases (22). Assessing the lung function of TB patients who successfully completed DOTS is important for understanding the extent of the impact of pulmonary impairment among the TB cases in the study area. Greater understanding of the extent of this allows to take appropriate measures in order to minimize this impact and improve quality of life for TB patients following their DOTS. This study aimed to examine post-TB lung function impairment and compare these with control participants with no history of TB.

## Materials and methods

### Study design and population

We conducted a community-based comparative cross-sectional study in Dale district, Sidama Region, South Ethiopia. Smear-positive TB (PTB+) patients who were successfully treated between the years 2010 and 2021 were included in the study. Dale was selected as the study district because it is a district with high rate of TB in Sidama. The principal investigator has done other related studies before in the study district (21, 23). The district has 36 rural kebeles (lowest administrative unit in Ethiopia). The kebeles included in the study were selected based on their high rates of smear-positive TB cases.

The post-TB participant group consists of PTB+ (sputum smear-positive or culture positive) patients who received successful treatment between the study period, who were over 18 years old, and not died nor migrated to other settings. The following patients were excluded from the post-TB participant group: smear negative TB cases; extra pulmonary TB cases; PTB+ patients who had visible chest deformities or surgeries, with neuromuscular disorders, or who were taking pulmonary medications except for COPD, and those who were taking cardiac medications during the data collection period. Pregnant women and patients with chronic illnesses that caused debilitation in the patients were also excluded from the study.

Control participants were over 18 years old and were matched with a corresponding TB patient by sex and ± 5 years of age, selected from right hand side of the fifth neighborhood house of the TB cases. The ratio of TB patients to controls was 1:1. Some of the same exclusion criteria were applied to both TB patients and controls. The following people were excluded from the control group: those with history of TB treatment; people with visible chest deformities like kyphosis, or who were taking pulmonary and/or cardiac medications during the data collection period.

### Sample size and sampling

In this study, we considered a 95% level of significance, 80% power, percent of exposure among the controls 10%, an Odds Ratio to be detected of 4.2, percent of unexposed with the outcome 3% and controls with cases ratio of 1:1, with 10% added for potential non response, we calculated a sample of 165 cases and 165 controls to be included in the study, however we included 167 cases and 156 controls in our analysis. Out of 36 rural kebeles in the study district, we selected the eight kebeles with the highest number of PTB+ cases. All PTB+ cases and controls in the selected kebeles that met the inclusion criteria were included in the study.

### Variables

Lung function impairment was the primary outcome measure in this study. It includes both obstructive and restrictive lung function impairment. The SGRQ COPD symptoms score was the secondary outcome measure. The independent variables were sociodemographic factors, body mass index (BMI), wealth index and behavioral characteristics, such as smoking and drinking alcohol.

### Data collection

The questionnaire was developed in English and then translated into local language, Sidamu Afoo. This instrument was piloted before data collection and corrections were made. Residents of the study communities, with at least a graduate level education in health science and fluency in both local languages, were trained to identify and interview the study participants. Data collectors training included explanation of the study objectives and interview techniques. Smear-positive PTB patients who received successful treatment during the study period were identified at primary health care units (health centers) in the study district. Patient name, care taker name, telephone number, address, kebele, age, sex and TB type (only PTB+ cases) were retrieved from the TB registry by each health center’s TB focal person. Then these TB patients were identified within their communities by data collectors. Controls were also recruited from the community. The data collector and guide identified the participants at home, interviewed them and instructed them to come to their local health post within two to 3 days for spirometry assessment. Data including date of completion of TB treatment, sociodemographic information, smoking history, weight, height, history of medically diagnosed chronic obstructive lung diseases (asthma, emphysema and chronic bronchitis), and patient reported symptoms of COPD were collected by record review and interview. Pre-testing was done on about 5% of the sample size on people located in another community a few days prior to the actual data collection. All the field work issues were carried out between 29/11/2022–24/02/2023. Supervision was conducted throughout the data collection period, and data was checked daily in order to ensure consistency and allow any problems encountered to be managed accordingly.

The ndd EasyOne spirometer, manufactured by ndd Medical Technologies (Switzerland), was used to measure lung function. The device is portable and has been used in other relevant studies (12, 24–26). We used a clean, reusable mouthpiece for every participant. The mouthpiece was soaked and cleaned in 10% chlorine solution after each use. Spirometry was performed in open air. Face masks, sanitizer and gloves were used to protect the enumerators. We measured the forced expiratory volume in 1 s (FEV_1_) and the forced vital capacity (FVC). A 1 min resting period was given between each manoeuvre. Three manoeuvres were performed to obtain the flow rate curve (FVC curve). The largest values for FEV_1_ and FVC from the three acceptable manoeuvres were retained to determine the final forced expiratory flow (FEF). The electronically-generated spirometric data, along with the patient ID, weight, height, and age of participant, were on paper and later entered into a computer database. The data collector explained and showed the technique to each of the study participants before they underwent the spirometry measurements. FEV1 was the amount of air that can forcibly be exhaled from the lungs in the first second of a forced exhalation. FVC is the volume of air that can forcibly be blown out after full inhalation. All lung function measurements were done by the principal investigator in consultation with a physiologist (YMW) who is a member of the research team. A member of the district health department, with second degree in a public health specialty, supervised the data collection process.

### Operational definitions

Restrictive pulmonary impairment is an impairment which causes restriction of airflow to the lungs. This was defined as having a normal FEV1/FVC ratio but low FEV_1_, below 3 litres. Obstructive pulmonary impairment is an impairment caused by obstruction of the airway and it was defined by an FEV1/FVC ratio below 70%. Having any impairment was calculated by having any of the impairment (restrictive pulmonary impairment or obstructive pulmonary impairment). A recent respiratory symptom was defined as a reported respiratory symptom, like cough or sputum, within the past 4 weeks. COPD symptoms were also assessed using the Saint Georges Respiratory Questionnaire (SGRQ).

The SGRQ was used to measure patient reported outcomes of COPD (27). It has been used in TB studies in the past. The symptoms component addresses the effect of respiratory symptoms, their frequency and severity. We used the symptoms component to evaluate the difference among our study participants. The score was expressed as a percentage of overall impairment where 100 represents worst possible symptom status and 0 indicates best possible symptom status (27).

### Data analysis

Data were analyzed using the SPSS statistical package version 25 (SPSS Inc., Chicago, IL, United States). Mean FEV_1_, FVC, FEV_1_/FVC ratio were calculated. To determine obstructive impairment, the main outcome measure; FEV1/FVC ratio, a continuous variable, was transformed into a categorical variable by designating the standard cutoff point 70%. This means participants with a ratio below 70% were considered to have obstructive lung impairment. Each participant’s FVC values were categorized into restrictive impairment, if the measurement was less than 3 liters with normal FEV1/FVC ratio. COPD symptom score was categorized in to low score indicating a better symptom status and high score indicating a bad symptom status. Low or high BMI was considered for a BMI score of below 18.5 kg/m^2^ or greater than 24.9 kg/m^2^. Wealth index was developed by considering house hold wealth related variables such as having household electricity, having any chair or table or bed, having radio, mobile, floor, roof, car-motor-cycle, land-for-agriculture, castles, toilet, income, and occupation. Then the final factor score was categorized in to low and high score by taking the median factor score as a cut-off point. Summary values were reported for the post-TB and control groups. Bivariate and multivariate logistic regression analyses were done to identify predictors of the outcome variables. Variables with *p*-value less than 0.2 in the bivariate logistic regression were included in the multivariate analysis. The strength of association between determinant factors and outcome variables was measured through odds ratios.

## Results

A total of 323 participants, (167 post-TB cases and 156 controls) were included in this study, with a response rate of nearly 97.9%. Ninety one (54.5%) post-TB participants and 92 (59.0%) controls were male. Concerning age, 92 (55.1%) post-TB participants and 107 (68.6%) controls were in the age group 18–45 years. Over half of both the post-TB (102 (61.1%)) and the control 87 (55.8%) groups had no formal education and 93 (55.7%) post-TB participants and 80 (51.3%) controls were farmers. Regarding wealth index scores, 90 (53.9%) post-TB patients and 71 (45.5%) controls had a low score. Over a quarter of the controls (43 (27.6%)), and 75 (44.9%) post-TB patients had either a low or high BMI. There was no statistically significant difference between the post-TB and the control groups for most of the sociodemographic variables. Nearly one-fifth of the post-TB group had been treated for TB at least twice. The median (interquartile range (IQR)) of the recent TB treatment completion was 24 (31.75) months for the TB cases (Table 1).

**Table 1:** Sociodemographic characteristics of the study participants.

Variables	Post-TB; *n* (%)	Controls; *n* (%)	Total; *n* (%)	*P*-value
Participants	167 (100)	156 (100)	323 (100)	
Sex				
Male	91 (54.5)	92 (59.0)	183 (56.7)	0.41
Female	76 (45.5)	64 (41.0)	140 (43.3)	
Age in years				
18–45	92 (55.1)	107 (68.6)	199 (61.6)	0.01
46–78	75 (44.9)	49 (31.4)	124 (38.4)	
Education				
No education	102 (61.1)	87 (55.8)	189 (58.5)	0.33
Has education	65 (38.9)	69 (44.2)	134 (41.5)	
Occupation				
Farmer	93 (55.7)	80 (51.3)	173 (53.6)	0.4
Housewife	59 (35.3)	53 (34.0)	112 (34.7)	
Student	13 (7.8)	20 (12.8)	33 (10.2)	
Other	2 (1.2)	3 (1.9)	5 (1.5)	
Wealth index				
Low score	90 (53.9)	71 (45.5)	161 (49.8)	0.13
High score	77 (46.1)	85 (54.5)	162 (50.2)	
BMI				
Low or high	75 (44.9)	43 (27.6)	118 (36.5)	0.001
Normal	92 (55.1)	113 (72.4)	205 (63.5)	
Number of TB treatment times				
Once	137 (82.0)		137 (42.4)	
Two or more	30 (18.0)		30 (9.3)	
Months since treatment completion; median (IQR)	24 (31.75)		24 (31.75)	

IQR, interquartile range; Low or high BMI: a BMI score < 18.5 or > 24.9 kg/m^2^, Normal BMI: 18.5–24.9 kg/m^2^.

A higher proportion of post-TB participants 9 (5.4%) than controls 2 (1.3%) had ever smoked cigarettes. Similarly, the proportion of khat chewing was higher among the post-TB participants 15 (9.0%) than among the controls 6 (3.8%). Moreover, alcohol drinking was higher in the post-TB group (15 (9.0%)) than in the control group (7(4.5%)). Nearly similar proportion of post-TB participants 3 (1.8%) and controls 3 (1.9%) used recreational drugs (Table 2). Based on our assessment using the SGRQ, the post-TB group had significantly higher symptoms scores than controls (see Table 3).

**Table 2:** Behavioral characteristics of participants with history of TB treatment and controls.

Variables	Post-TB; *n* (%)	Controls; *n* (%)	Total; *n* (%)	*P*-value
Smoking				
Yes	9 (5.4)	2 (1.3)	11 (3.4)	0.04
No	158 (94.6)	154 (98.7)	312 (96.6)	
Khat chewing				
Yes	15 (9.0)	6 (3.8)	21 (6.5)	0.06
No	152 (91.0)	150 (96.2)	302 (93.5)	
Alcohol				
Yes	15 (9.0)	7 (4.5)	22 (6.8)	0.1
No	152 (91.0)	149 (95.5)	301 (93.2)	
Recreational drug using				
Yes	3 (1.8)	3 (1.9)	6 (1.9)	0.9
No	164 (98.2)	153 (98.1)	317 (98.1)	

**Table 3:** The lung function related characteristics of the study participants.

Variables	Post-TB; *n* (%)	Controls; *n* (%)	Total; *n* (%)	*P*-value
Symptom score				
Low score	14 (8.4)	136 (87.2)	150 (46.4)	< 0.001
High score	153 (91.6)	20 (12.8)	173 (53.6)	
AWO				
No	66 (39.5)	93 (59.6)	159 (49.2)	< 0.001
Yes	101 (60.5)	63 (40.4)	164 (50.8)	
COPD by interview				
No	83 (49.7)	151 (96.8)	234 (72.4)	< 0.001
Yes	84 (50.3)	5 (3.2)	89 (27.6)	
Resent respiratory symptoms				
Yes	123 (73.7)	9 (5.8)	132 (40.9)	< 0.001
No	44 (26.3)	147 (94.2)	191 (59.1)	

AWO, airway obstruction; COPD, chronic obstructive pulmonary diseases.

The overall lung function in the post-TB group was worse than in the control group (see table 4). Notably obstructive impairment appears to be more common among the post-TB participants than restrictive impairment. High proportion of post-TB participants 101 (60.5%) than controls (40.4%) had obstructive impairment, *p* = 0.01. Over one-fifth (22.8%) of the post-TB participants and 39 (25.0%) controls had restrictive impairment; *P* = < 0.3.

**Table 4:** Pulmonary function of post-TB participants and controls in Sidama, South Ethiopia.

Parameters	Post-TB, *n* = 167	Controls, *n* = 156	Estimate* (95% CI) *P*-value
FEV1 (mean/SD)	1.80 ± 0.81	2.29 ± 0.97	−0.49 (−0.69-(−0.29)) <0.001
FVC (mean/SD)	2.89 ± 1.06	3.35 ± 1.22	−0.46 (−0.71-(−0.21)) <0.001
FEV1/FVC (mean/SD)	62.23 ± 15.65	69.83 ± 19.19	−7.6 (−11.5-(−3.8)) <0.001
Obstructive impairment, *n* (%)	101 (60.5)	63 (40.4)	2.3 (1.5–3.5) 0.01
Restrictive impairment, *n* (%)	38 (22.8)	39 (25.0)	0.9 (0.5–1.5) 0.3
Any impairment, *n* (%)	139 (83.2)	102 (65.4)	2.6 (1.6–4.5) < 0.001

FEV1, forced expiratory volume in 1 min; FVC, forced vital capacity; FEV1/FVC, the ratio of forced expiratory volume in 1 min and forced vital capacity; SD, standard deviation; CI, confidence interval; Estimate* = mean difference or odds ratio.

In a bivariable and multivariable analysis; BMI score showed statistically significant association with obstructive pulmonary impairment. Participants with history of PTB+ had increased risk of obstructive pulmonary impairment (aOR, 2.1; 95% confidence interval (CI), 1.3–3.3). Participants with high or low BMI score also had increased risk of obstructive pulmonary impairment (aOR, 1.6; 95% CI, 1.0–2.6). Based on our analysis, none of the variables measured showed association with restrictive pulmonary impairment. However, being treated for TB (aOR, 2.2; 95% CI, 1.1–4.5) was associated with having any lung function impairment. In our assessment using the SGRQ, being treated for TB showed a statistically significant association with a high symptoms score (aOR, 73.0; 95% CI, 35.3–151.2).

## Discussion

In this comparative, cross-sectional study, a higher proportion of post-TB participants had obstructive lung impairment compared with the controls. This trend was also observed in the prevalence of obstructive symptoms. However, it is noteworthy the large proportion of controls with restrictive pulmonary impairment. There was no association between being treated for TB and restrictive pulmonary impairment. Being treated for TB was significantly associated with both obstructive lung impairment and having any type of lung impairment. It was also found to have a statistically significant association with the symptom score of the SGRQ. Being treated twice for TB did not predict lung function impairment (Table 5).

**Table 5:** Risk factors of pulmonary impairment among the study participants.

Outcome	Obstructive impairment		High symptom score	
Variables	COR (95% CI)	AOR (95% CI)	COR (95% CI)	AOR (95% CI)
Post-TB participants	2.3 (1.5–3.5)	2.1 (1.3–3.3)	74.3 (36.1–152.8)	73.0 (35.3–151.2)
BMI (high or low)	1.8 (1.2–2.9)	1.6 (1.0–2.6)	1.9 (1.2–3.0)	1.1 (0.5–2.4)

Outcome	Any one of impairment	
Variables	COR (95% CI)	AOR (95% CI)
Post-TB participants	2.6 (1.6–4.4)	2.2 (1.1–4.5)
Respiratory symptoms	2.3 (1.3–4.0)	1.3 (0.6–2.8)

COR, crude odds ratio, AOR, adjusted odds ratio, CI, confidence interval, Low or high BMI: a BMI score < 18.5 or > 24.9 kg/m^2^, Normal BMI: 18.5–24.9 kg/m^2^.

Regarding respiratory symptoms, this study revealed that a higher proportion TB patients had recent respiratory symptoms compared to the control group. Also, the post-TB participants had an increased risk of high COPD symptoms score. The respiratory symptoms were assessed using both the SGRQ and interviews, which inquired about symptoms such as cough or sputum. This finding aligns with the study from China, which also observed a higher proportion of respiratory symptoms among post-TB participants (28).

Respiratory obstruction in the current study was defined, as it is in many studies, as an FEV1/FVC ratio of <70%. However, by this definition 40% of the controls had obstructive lung disease having their mean FEV1/FVC of less than 70%. Though the difference in prevalence compared to post TB cases is statistically significant, the indicator was too high. Perhaps this relates to the universal exposure of participants to biomass combustion products among the post TB cases or controls as the socioeconomic status of our study participants was generally low. Our data showed that, over half (53.9%) of the TB cases and 45.5% of controls had low wealth index score and there is no statistically significant difference between cases and controls on wealth index.

The pulmonary impairment observed among post-TB participants in Dale was higher than that reported in Moscow, Russia (13). The main type of impairment observed among the post-TB participants was obstructive impairment. Over 60 % of post-TB participants had obstructive airway impairment, which was higher than the prevalence observed among the control group (40.4%). This figure was also higher than that reported in Russia (34.6%), China (21.3%) and Korea (30.3%) (12–14, 28). In Latin America, the overall prevalence of airflow obstruction was 30.7% among those with a history of TB, compared with 13.9% among those without (12). The differences in these prevalence figures could be related to the difference in types of TB. Bacteriologically confirmed TB cases included in this study might have more lung damage than other types of TB. As nearly one-fifth of the post-TB cases had history of TB treatment more than once, that may also contribute for the observed differences in pulmonary impairment. Recurrent TB could also result in more lung damage and it may result in more COPD symptoms.

Recurrent TB may increase the damage of lung tissue resulting an increased risk of lung function impairment. Hnizdo et al. (29) in their studies reported that tuberculosis can cause impairment of lung function which increases incrementally with the number of episodes of tuberculosis. However, the data in the current study did not confirm such an association. This could be related to having low proportion of TB cases with recurrent TB.

In the current study, more cases than controls had low or high BMI score and having an abnormal BMI increased the risk of pulmonary impairment. This could be due to the presence of low respiratory muscle bulk and pathological conditions in people with low or high BMI. Consistent to the current study finding, certain report confirmed that obesity was associated with lower FEV_1_ and FVC and there was a decrease in FVC in participants with central obesity showing the association between BMI and lung function is dependent on the presence of central obesity (30). Bhatti et al. (31) in their study found a significant association between the BMI and pulmonary function parameters as obesity causing detrimental effects on respiratory function. Also, being underweight and severely obese were associated with reduced lung function according to the report by Tang et al. (32). This association could be explained by underweight people may have nutritional deficiency, and low muscle mass (33), which may have a negative effects on the lung function (34, 35).

Long-term effects of TB include inflammation and destruction of the lung parenchyma, which causes scarring and fibrosis (8). This may lead to restrictive pulmonary impairment. The proportion of post-TB participants with restrictive pulmonary impairment was lower than in the control group and the report from another setting (13). In Russia, 28.0% of TB patients had reduced pulmonary function (FEV_1_ below the lower limit of normal) (13). This difference could be explained by many controls involved in this study having other diseases which affect the lung parenchyma. Over a third (36.4%) of the controls reported a recent cough episode during the data collection period. Another possible reason for the difference could be lower air pollution in rural Sidama compared with urban China and Russia (13, 28). Another possible reason for these differences could be the low number of smokers included in this study; only 5.4% were smokers. Whereas, smokers made up 64.5% of the participants in Moscow, Russia, (13) and 33.6% in China (28). We suggest continuing care for TB patients after completing TB treatment to minimize the impacts of pulmonary impairment.

The FEV_1_/FVC% was significantly decreased in subjects with prior PTB compared to those without in another study (19). Other studies too confirmed the association between a history of TB and the presence of chronic airway obstruction (10, 13, 18, 19, 28). Similarly, the current study also found participants with a history of TB had an increased risk of pulmonary impairment (10, 13, 18, 19, 28). Providing prevention measures such as smoking cessation, pollution control, and timely identification and management of pulmonary impairment among the TB cases who completed treatment may be helpful to minimize the long-term effects of TB.

Our study was conducted in rural Sidama, Ethiopia. Households in rural Ethiopia mainly use wood for cooking and most of them have no separate kitchen. The proportion of households using solid fuel in Ethiopia is 94.03% (36). In line with this report, nearly all our study participants (97.3%) used wood for cooking. Over 96% of the study participants were non-smokers and majority (55.1%) had no separate kitchen. This could be the cause of high restrictive impairment in controls and worse symptoms on post TB compared to other settings. However, none of these factors were significantly associated with COPD in the current study. Therefore, the association between a history of TB and obstructive lung function impairment was not affected by these factors. A multi-centre LMICs study also reported an increased risk of COPD among people with a history of TB (18). This could be due to airway damage, bronchiolar narrowing, bronchiolitis or emphysematous change, caused by chronic or recurrent inflammation, leading to COPD as reported in other studies.

This is the first study of its kind reported from Ethiopia which has a population of over 120 million, with high incidence of TB. To control confounding effect, post-TB participants and controls were matched according to age and sex and we performed an adjusted analysis for variables that fulfilled the inclusion criteria for an adjusted analysis. However, we did not collect data on variables like having of infections like HIV, pneumocystis carnie pneumonia (PCP) and having of post-COVID Lung disease. So, we did not control their effects in our analysis. This could be considered among the limitations of this study. The other limitations observed in this study was the sample size; which was inadequate for measuring the association between a history of TB and respiratory obstruction symptoms. The 95% confidence intervals for some of the symptom component of the SGRQ was wide. However, the sample size we used to estimate pulmonary impairment was quite adequate. Our study participants were homogeneous in some characteristics, like smoking and use of biomass fuel. Due to lack of variation, we were unable to see the effect of these variables on pulmonary impairment. Another limitation of this study is a potential survivor bias. Some patients who were successfully treated for TB during the study window died before interview; these deaths could have been caused by COPD (37). Therefore, this could have created a survivor bias in the study. The other limitation of the current study is that we used SGRQ symptom scores to measure patient reported outcomes of COPD. The SGRQ assess participant’s quality of life, not disease severity in principal. More robust markers such as percentage predicted FEV1, COPD Assessment Test (CAT) scores, Modified Medical Research Council (mMRC) scores and 6 min walk test would have been used to measure the severity. The use of these parameters could improve on the rigor of the outcome variables. Since we did not collect data on these parameters, we were not able to use them. Authors of this study suggest readers to take in to consideration of the aforementioned limitations while interpreting the findings.

## Conclusion

Our study showed that people with a history of TB had a higher rate of obstructive pulmonary impairment and respiratory obstructive symptoms compared to demographically similar control participants. Post-TB participants had an increased risk of obstructive lung impairment and high COPD symptoms scores, but no increased risk of restrictive pulmonary impairment. Having a high or low BMI also increased the risk of pulmonary impairment.

We suggest continuing care for TB patients after completion of DOTS. Providing primary prevention measures such as smoking cessation, pollution control and timely identification and management of pulmonary impairment could help to limit long-term impacts of TB. Assessment of the COPD symptoms and the pulmonary function could be done 6 months after completion of treatment among TB patients to aid early identification of and provide timely management for pulmonary impairment. Post-TB COPD early identification and treatment of COPD symptoms might benefit to improve quality of life and relieve symptoms in this group of population.

## Data Availability

The raw data supporting the conclusions of this article will be made available by the authors, without undue reservation.
